# Empiric versus imaging guided left ventricular lead placement in cardiac resynchronization therapy (ImagingCRT): study protocol for a randomized controlled trial

**DOI:** 10.1186/1745-6215-14-113

**Published:** 2013-04-26

**Authors:** Anders Sommer, Mads Brix Kronborg, Steen Hvitfeldt Poulsen, Morten Böttcher, Bjarne Linde Nørgaard, Kirsten Bouchelouche, Peter Thomas Mortensen, Christian Gerdes, Jens Cosedis Nielsen

**Affiliations:** 1 Department of Cardiology, Aarhus University Hospital, Brendstrupgaardsvej 100, Skejby DK-8200, Aarhus N, Denmark; 2 Department of Nuclear Medicine, Aarhus University Hospital, Brendstrupgaardsvej 100, Skejby DK-8200, Aarhus N, Denmark

**Keywords:** Heart failure, Cardiac resynchronization therapy, Cardiac imaging, Left ventricular lead placement

## Abstract

**Background:**

Cardiac resynchronization therapy (CRT) is an established treatment in heart failure patients. However, a large proportion of patients remain nonresponsive to this pacing strategy. Left ventricular (LV) lead position is one of the main determinants of response to CRT. This study aims to clarify whether multimodality imaging guided LV lead placement improves clinical outcome after CRT.

**Methods/Design:**

The ImagingCRT study is a prospective, randomized, patient- and assessor-blinded, two-armed trial. The study is designed to investigate the effect of imaging guided left ventricular lead positioning on a clinical composite primary endpoint comprising all-cause mortality, hospitalization for heart failure, or unchanged or worsened functional capacity (no improvement in New York Heart Association class and <10% improvement in six-minute-walk test). Imaging guided LV lead positioning is targeted to the latest activated non-scarred myocardial region by speckle tracking echocardiography, single-photon emission computed tomography, and cardiac computed tomography. Secondary endpoints include changes in LV dimensions, ejection fraction and dyssynchrony. A total of 192 patients are included in the study.

**Discussion:**

Despite tremendous advances in knowledge with CRT, the proportion of patients not responding to this treatment has remained stable since the introduction of CRT. ImagingCRT is a prospective, randomized study assessing the clinical and echocardiographic effect of multimodality imaging guided LV lead placement in CRT. The results are expected to make an important contribution in the pursuit of increasing response rate to CRT.

**Trial registration:**

Clinicaltrials.gov identifier NCT01323686. The trial was registered March 25, 2011 and the first study subject was randomized April 11, 2011.

## Background

Cardiac resynchronization therapy (CRT) is an established treatment in symptomatic heart failure patients with depressed left ventricular (LV) ejection fraction (EF) and prolonged QRS duration [1-5]. The therapy is implemented by implanting a device with three pacing leads (one in the right atrium, one in the right ventricle, and one in a LV epicardial vein) that uses atrial-synchronized biventricular pacing to coordinate RV and LV contraction. Despite technical advances and increasing experience in CRT, approximately 30 to 50% of patients derive no clinical benefit from this treatment [1,2,4]. An important determinant of response to CRT is selection of the LV pacing site [6,7]. Currently, the LV lead is preferably placed in a lateral or postero-lateral branch of the coronary sinus (CS) [8]. Retrospective studies have shown superior clinical and echocardiographic outcome when placing the LV lead in a region without myocardial scar and concordant to the site of the latest mechanical activation [6-13]. Assessment of the latest activated LV myocardial segment, scar areas and cardiac venous anatomy can be performed by speckle tracking echocardiography, single-photon emission computed tomography (SPECT), and cardiac computed tomography (CT), respectively [7,9,12,14,15]. Promising results on CRT outcome have been reported in recent prospective studies evaluating the effect of echocardiography guided LV lead placement [16,17].

To clarify the potential role of imaging guided LV lead positioning we have designed a prospective, patient- and assessor-blinded, randomized trial comparing empiric versus multimodality imaging guided LV lead placement on clinical outcome after CRT. The main objective of this study is to determine the effect of imaging guided LV lead placement (targeted to the latest activated non-scarred myocardial region by speckle tracking echocardiography, SPECT and cardiac CT) on clinical response rate to CRT.

## Methods

### Study design

**Figure 1:**
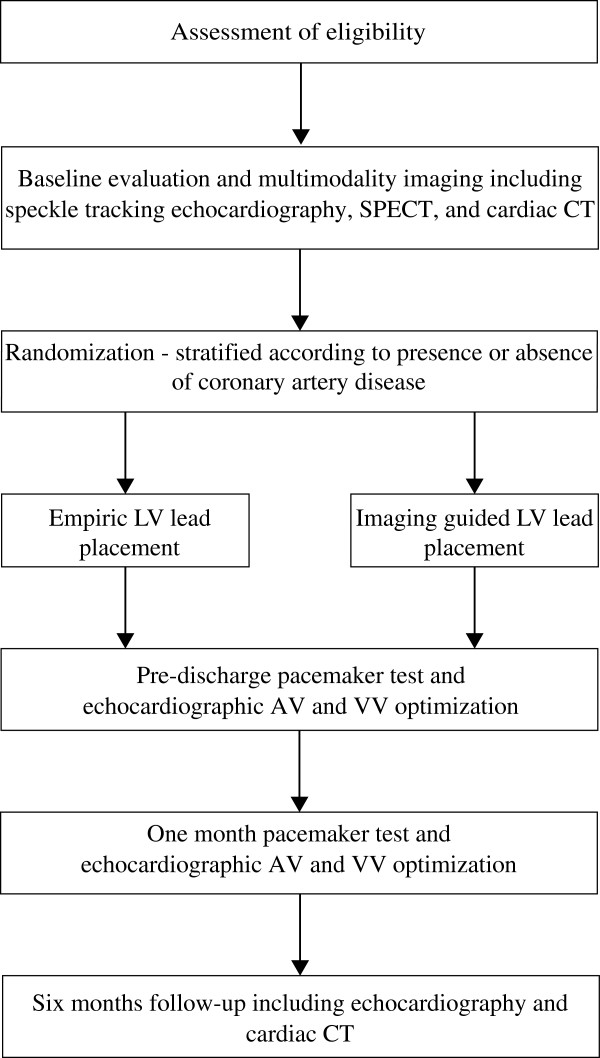
**Study design.** Flow chart describing the ImagingCRT study design. Randomization is stratified according to presence or absence of coronary artery disease. AV, atrioventricular; CT, computed tomography; LV, left ventricle; SPECT, single-photon emission computed tomography; VV, interventricular.

The ImagingCRT study is a prospective, patient- and assessor-blinded, randomized, single center trial with two study arms. Echocardiography, SPECT and cardiac CT is performed in all study participants. Subsequently, patients are randomly assigned to standard LV lead placement preferably in a lateral or posterior-lateral position or LV lead positioning guided by multimodality imaging to the latest mechanically activated non-scarred myocardial region. Patients are allocated 1:1 to each intervention arm and followed for six months (Figure 1). A total of 192 patients will be enrolled in the study.

### Study population

Study subjects are recruited at a tertiary referral center (Department of Cardiology, Aarhus University Hospital, Skejby, Denmark). Study enrollment criteria are listed in Table 1. Patients receiving a CRT pacemaker (CRT-P) or a CRT-implantable cardioverter defibrillator (CRT-D) device are eligible for enrollment. Patients undergoing upgrade from a single-lead or dual-chamber pacemaker or implantable cardioverter-defibrillator can be included. Reasons for excluding patients are recorded.

**Table 1:** Study enrollment criteria

***Inclusion criteria***	***Exclusion criteria***
• NYHA functional class II to IV despite optimal medical treatment	• Recent myocardial infarction (<3 months)
• ECG with QRS >120 ms and LBBB configuration or paced QRS >180 ms	• Expected lifetime <6 months
• LV EF ≤35%	• Pregnant or lactating
• Age >40 years	• Inadequate echocardiographic images for determination of site with latest mechanical activation
• Written informed consent	• No written informed consent

Inclusion/exclusion criteria. LBBB: left bundle branch block; LV EF: left ventricular ejection fraction; NYHA: New York Heart Association [20].

### Ethical considerations

The study conforms to the principles outlined in the Declaration of Helsinki [18]. The Central Denmark regional committee on health research ethics (file no. 20100245) and the Danish Data Protection Agency (file no. 2011-41-6017) have approved the study protocol. Patients participate in the study only after giving informed written consent.

### Data collection and recordings

All study data are recorded in a web based case record form (eCRF) with logging of all data entries. All investigators have access to the eCRF.

## Assessment of study variables

### Baseline clinical and functional evaluation

Medical records are assessed. Functional capacity is evaluated using the six-minute-walk test (6MWT) [19] and the New York Heart Association (NYHA) classification [20]. Quality of life is assessed by the Minnesota Living with Heart Failure Questionaire, a 21-item disease specific instrument with scores varying from 0 to 5 and a summary score varying from 0 to 105, the highest score representing the worst health-related quality of life. Validity of the questionaire has been established and a Danish version exists [21-23].

Blood is drawn for laboratory test of renal function and N-terminal pro b-type natriuretic peptide (NT-ProBNP).

### Echocardiography

Baseline and follow-up echocardiographic images are obtained at rest with a 3.5-MHz transducer using a commercially available system (Vivid E9, GE Medical Systems, Horten, Norway). Two-dimensional (2D), color Doppler and tissue Doppler imaging (TDI) data triggered to the ECG are stored in cine-loop format and transferred to a workstation for offline analysis using dedicated software (EchoPac BT11, GE Medical Systems, Horten, Norway). At least three consecutive beats are recorded during breath hold in all views. All measurements are averaged over three cycles.

LV end-diastolic volume (EDV) and end-systolic volume (ESV) are assessed from standard apical four- and two-chamber views. LV EF is estimated using Simpson’s biplane method [24]. Severity of mitral regurgitation is graded semi-quantitatively [25].

### LV dyssynchrony and latest activated myocardial region

**Figure 2:**
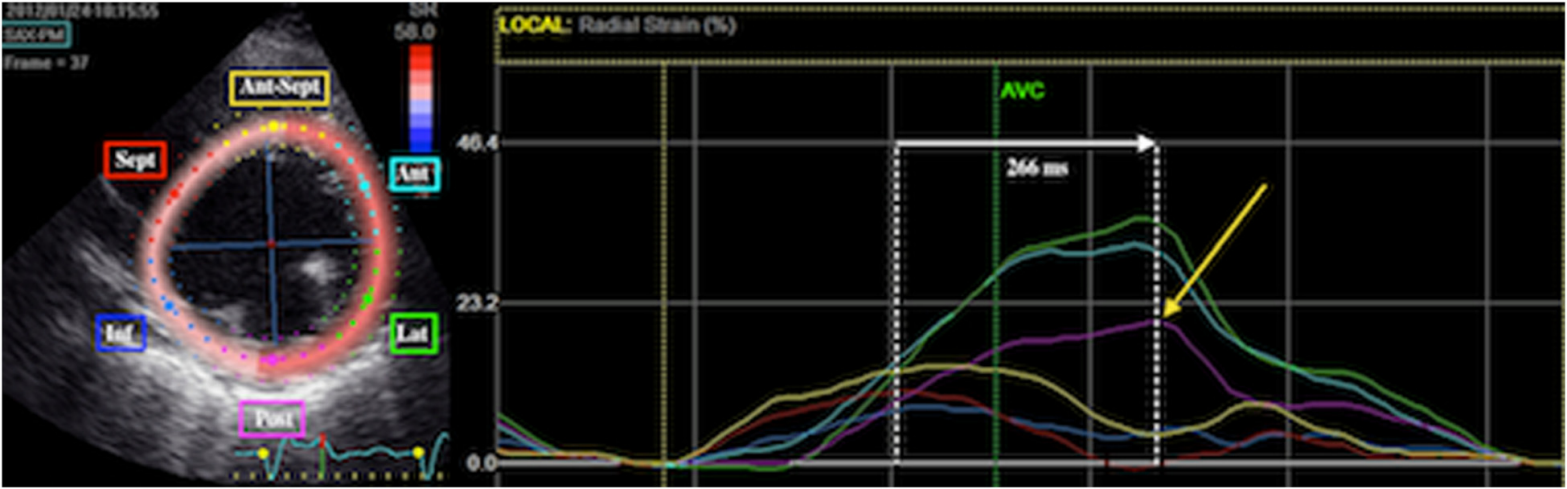
**Speckle tracking radial strain analysis.** Assessment of LV radial dyssynchrony and the latest mechanically activated segment by two-dimensional speckle tracking radial strain imaging. Time-strain curves are shown for the six mid-LV segments. This patient example illustrates LV dyssynchrony with a 266 ms delay between the septal and posterior segment. Site of the latest mechanical activation is the posterior segment (yellow arrow). Ant, anterior; Ant-Sept, antero-septal; Inf, inferior; Lat, lateral; Post, posterior; Sept, septum.

LV dyssynchrony is evaluated using 2D speckle tracking radial strain on mid-LV short axis views [26]. Images are obtained with a frame rate of 50 to 80 s^-1^. Care is taken to avoid oblique mid-LV short axis views and to obtain images with the most circular geometry. In an end-systolic frame, the LV endocardial contour is traced. Subsequently, the software automatically defines the region of interest including the entire myocardial wall. Adequate tracking of the region of interest throughout the cardiac cycle is ensured by visual control and, if necessary, optimized by manual adjustment. Time-strain curves are generated for the six mid-LV segments (antero-septal, anterior, lateral, posterior, inferior and septal). Time from QRS onset to peak radial strain is measured in all segments and the latest activated segment is identified. LV dyssynchrony is measured as time delay between the earliest and latest activated segment (Figure 2) [27].

In a small minority of patients we expect that speckle tracking is not feasible. In that case we use tissue synchronization imaging with automated color-coding of time to peak longitudinal velocities derived from TDI to identify the mid-LV segment with latest activation [11,15,28].

### Localization of myocardial scar

**Figure 3:**
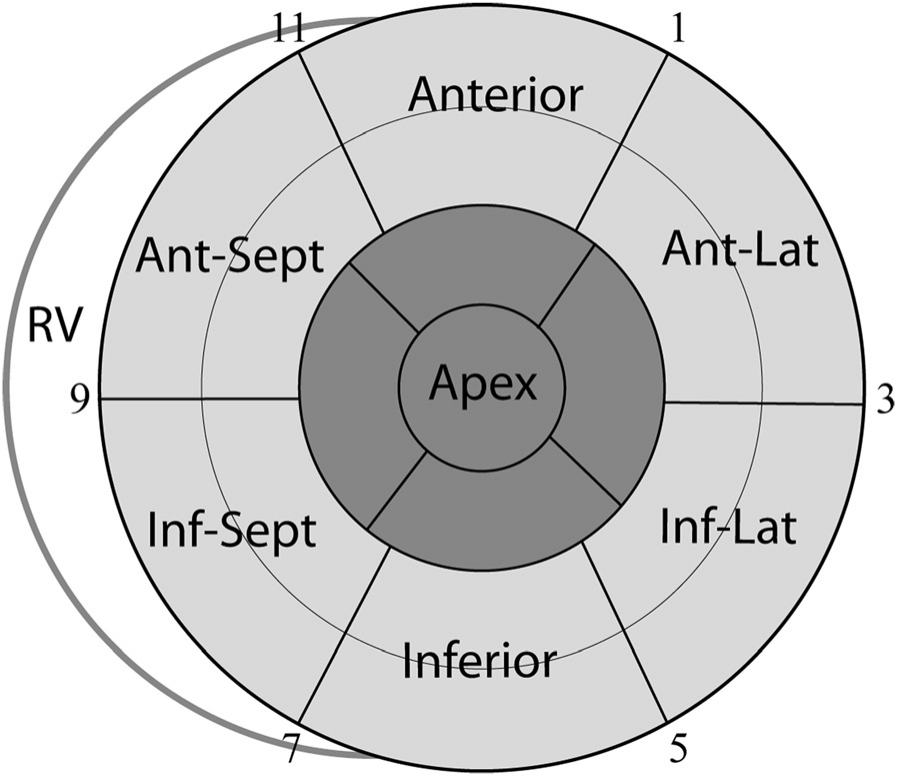
**Left ventricular myocardial segmentation.** The LV 17-segment model used for SPECT interpretation. Basal and mid-LV segments depicted in light gray are considered equal for LV lead placement. Apical segments and apex are depicted in dark gray. Numbers are in accordance with the clockwise method used for the fluoroscopic venogram in the left anterior oblique projection. The antero-lateral, infero-lateral and infero-septal segments correspond to the echocardiographic lateral, posterior and septal segments, respectively. Ant-Lat, antero-lateral; Inf-Lat, infero-lateral; Inf-Sept, infero-septal; RV, right ventricle.

Rest ^99m^Tc-sestamibi SPECT (700 ± 70 MBq) is performed 60 minutes after radiotracer injection using a dual-headed rotating gamma camera (CardioMD, Phillips Healthcare, Andover, MA, USA) with a high-resolution, parallel-holed collimator. Sixty-four projections of 25 seconds are acquired over a non-circular 180° arc. Images are gated with eight frames per cardiac cycle. Analysis and reconstruction of the acquired data are performed using commercially available software (AUTOSPECT, AUTOQUANT; ADAC Laboratories, Milpitas, CA, USA). In case of failure of the automatic algorithm, manual tools for masking extra cardiac activity or defining the valve plane and the apex of the left ventricle are used. Data are displayed in polar map format (normalized to maximal tracer activity) and analyzed using the 17-segment model (Figure 3) [29]. A tracer uptake ≥75% is considered normal myocardium, 50 to 75% tracer uptake is considered non-transmural scar tissue, and an uptake <50% is considered transmural scar tissue [9].

### Assessment of cardiac venous anatomy and LV lead position

Cardiac CT is performed using a second-generation dual-source CT system (Siemens Somatom Definition Flash, Siemens Healthcare, Erlangen, Germany). Cardiac CT is performed with 80 to 120 kV tube voltage, adaptive tube current with a reference of 370 mAs, 128 × 0.6 mm collimation, z-flying spot and a gantry rotation time of 280 msec. In retrospective scans an ECG-controlled tube current modulation is applied with reduction of the current to 20% and full pulsing applied only from 60 to 70% of the RR interval. Retrospective scan pitch is 0.2 to 0.4 depending on heart rate, and 3.4 for prospective scans, respectively. An iterative reconstruction algorithm is applied (SAFIRE, Siemens Healthcare, Erlangen, Germany).

**Figure 4:**
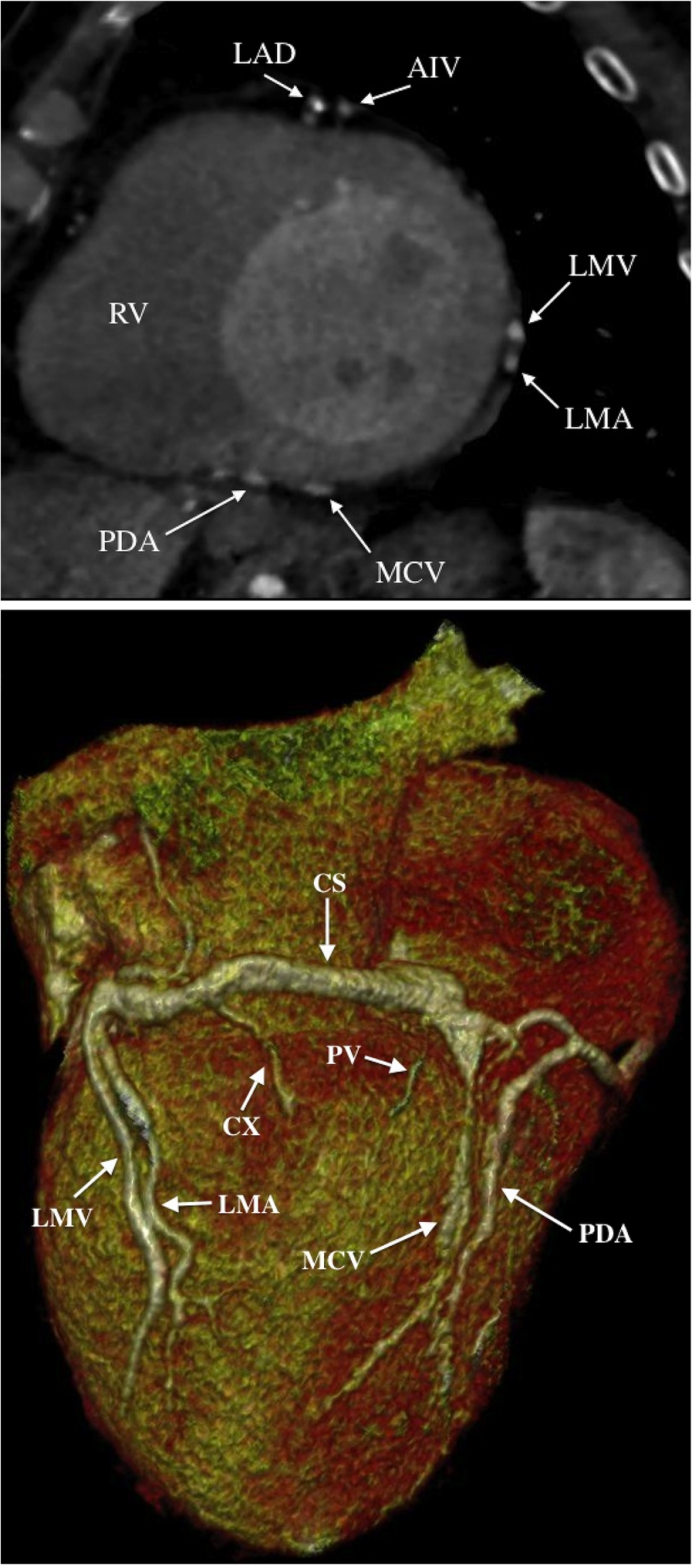
**Evaluation of cardiac venous anatomy.** Baseline cardiac CT images from a prospective high pitch scan illustrating cardiac venous anatomy in a chronic heart failure patient in the mid-LV multiplanar reformatted short axis view (top) and in a three-dimensional volume-rendered reconstruction (bottom). AIV, anterior interventricular vein; CS, coronary sinus; CX, circumflex coronary artery; LAD, left anterior descending artery; LMA, left marginal artery (circumflex artery branch); LMV, left marginal vein; MCV, middle cardiac vein; PDA, posterior descending artery (right coronary artery branch); PV, posterior vein (PV and CX are not seen in the mid-LV short axis view); RV, right ventricle.

A two-step scan protocol is performed at baseline. An ECG triggered test bolus is used to capture the CS with regions of interest placed in the proximal CS and in the LV cavity. During breath hold, image acquisition is achieved in two consecutive steps. First, a retrospective ECG-gated scan timed according to optimal contrast filling in the LV cavity. Subsequently, a prospective ECG-gated high-pitch scan corresponding to optimal contrast filling in the proximal CS. Commercially available contrast media (Optiray® 350 mg/ml, Covidien, Hazelwood, MO, USA) is used (20 ml for the test bolus and 50 to 60 ml for the two-step scan). Contrast injection is followed by a 50 ml saline flush. Images are analyzed using commercially available software (Syngo.via and Multimodality workplace, Siemens Healthcare, Erlangen, Germany). The observer individually adjusts window settings. For evaluation of cardiac venous anatomy, axial, multiplanar reformats and three-dimensional (3D) images are reconstructed (Figure 4).

**Figure 5:**
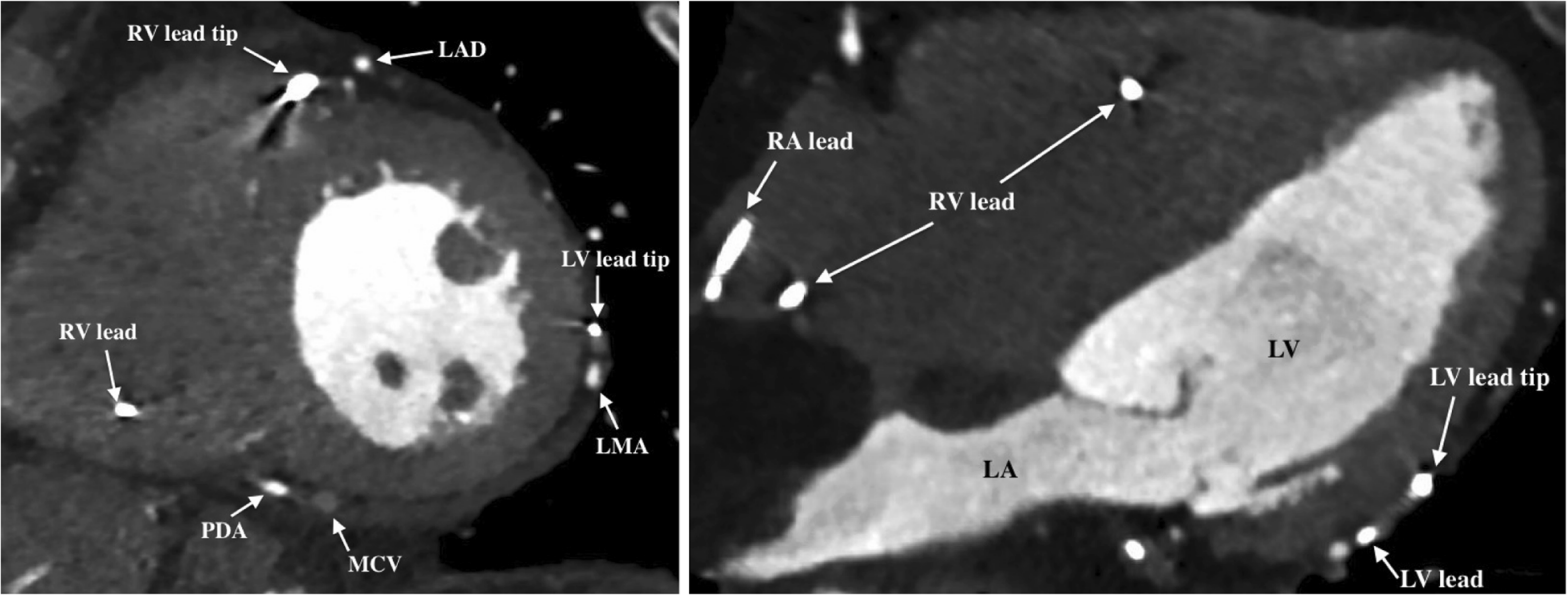
**Evaluation of LV lead position.** Six months follow-up cardiac CT acquisition from the same patient as in Figure 4. In the mid-LV short axis view, the LV lead is positioned in the left marginal vein draining the lateral LV wall (left). In the four-chamber view, the LV lead tip is positioned in the lateral mid-LV segment (right). LA, left atrium; LAD, left anterior descending artery; LMA, left marginal artery (circumflex artery branch); MCV, middle cardiac vein; PDA, posterior descending artery (right coronary artery branch); RA, right atrium; RV, right ventricle).

Six months after CRT implantation, a repeat retrospective contrast enhanced ECG-gated CT scan is performed for evaluation of LV lead position (Figure 5).

In a small minority of patients with an allergy to contrast media or severely depressed renal function (estimated glomerular filtration rate <30 ml/min) baseline cardiac CT is omitted. Then, only the pre-implant coronary venography is used for visualization of cardiac venous anatomy and a non-contrast CT scan is performed for LV lead localization at six months follow-up.

### Optimal LV pacing site

The optimal LV pacing site is defined as the myocardial region with the latest mechanical activation and without transmural scar tissue. Basal and mid-LV segments in the same LV wall are considered equally optimal (Figure 3). Combining information from speckle tracking echocardiography, SPECT and cardiac CT, imaging guided LV lead placement is targeted to a vein at the optimal pacing site. When two segments are equally delayed, both segments are considered optimal for LV pacing. If the latest activated segment has a SPECT tracer uptake <50%, the second latest activated segment is chosen as the optimal pacing site. CT images of cardiac venous anatomy are assessed in 2D mid-LV short axis views corresponding to a fluoroscopic left anterior oblique (LAO) projection and in three-dimensional volume-rendered reconstructions (Figure 4). CS tributaries closest to the optimal LV pacing site are labeled as first priority for LV lead placement. Tributaries closest to the second, third or fourth latest activated segment with viable myocardium are chosen as second, third or fourth priority, respectively. Images illustrating the latest activated segment, the distribution of scar tissue, and the prioritized CS tributaries are available to the implanting physician *only* when the patient is randomized to imaging guided LV lead placement.

### CRT implantation

All leads are implanted transvenously using commercially available leads and devices. The right atrial lead is placed in the right atrial appendage. In both CRT-P and CRT-D implantations, the right ventricular lead is placed on the interventricular septum or in the apex if a septal position is not reached. CS venograms are obtained before implantation of the LV lead. In the LAO 40 to 60° projection, the CS with its tributaries is seen encircling the mitral valve ostium. During implantation the optimal LV pacing site is located by the clockwise method in the LV short-axis circumference [30]. According to the echocardiographic LV short-axis view and the SPECT polar map, the LV is divided into six equal segments from the venogram in the LAO projection: Anterior-septal (9 to 11 o’clock), anterior (11 to 1 o’clock), lateral (1 to 3 o’clock), posterior (3 to 5 o’clock), inferior (5 to 7 o’clock), and septal (7 to 9 o’clock) (Figure 3). If the patient is randomized to imaging guided LV lead placement, the implanter aims to place the LV lead in the vein closest to the optimal LV pacing site. If the primary vein is not reached, the CS tributaries are aimed in prioritized order until a stable position is reached with acceptable thresholds below 2.5 V and with no phrenic nerve stimulation at an output less than twice the threshold. Empiric LV lead positioning is targeted to the lateral or postero-lateral region, preferably in an area with late electrical activation as measured online (CardioLab IT, GE Healthcare, Waukesha, WI, USA). Visualized in the right anterior oblique (RAO) 20 to 40° view the LV lead is placed in a midventricular or basal position avoiding apical segments.

### Follow-up

The device is tested at day one, one month and six months after implantation to ensure sufficient biventricular pacing (>95% of the time). Atrioventricular (AV) and interventricular (VV) optimization is performed at day one and repeated after one month. Optimal AV delay is obtained by the iterative method using transmitral pulsed wave Doppler to achieve the longest LV filling time with adequate E- and A-wave separation and termination of the A-wave. Optimal VV delay is programmed by changing the V-V sequence measuring the highest LV outflow tract time-velocity integral [28].

At six months follow-up, clinical, functional and echocardiographic variables are assessed as performed at baseline. LV lead position is assessed by cardiac CT as described and categorized as concordant (located at the optimal LV pacing site), as adjacent (one segment away) and remote (≥2 segments away from the optimal LV pacing site).

### Endpoints

The primary endpoint is non-response to CRT defined as the combination of all-cause mortality, hospitalization for heart failure, and unchanged or worsened functional status. The primary endpoint is attained if the patient is registered for one of the following three events during follow-up: (1) death from any cause, (2) hospitalization for heart failure, or (3) no improvement in NYHA class *and* <10% improvement in 6MWT.

Hospitalization for heart failure is defined as admission to the hospital lasting more than 24 hours with symptoms of congestive heart failure and subsequent intravenous treatment for heart failure.

Secondary endpoints include all-cause mortality, hospitalization for heart failure and changes of the following variables during follow-up: NYHA class, 6MWT, quality of life, Nt-ProBNP, LV EF, LV EDV and ESV, LV dyssynchrony and mitral regurgitation grade. Also, procedure time for CRT implantation, ability to place the LV lead in the first or second prioritized CS branch, complications and final lead positions are recorded.

### Sample size

The study is designed to detect a clinically meaningful difference between standard and imaging guided LV lead positioning in CRT as measured by applying a clinical composite endpoint. The underlying assumptions for the primary endpoint are based on data from previous trials in comparable patient cohorts demonstrating a 50 to 70% response rate to CRT [1,2,4,30]. Accordingly, we expect a 60% response rate in the group with empiric LV lead placement and hypothesize an 80% response rate in the group with imaging guided LV lead positioning. Thus, we estimate a 20% difference in the percentage of patients reaching the primary endpoint. To identify this increase in response rate and to achieve a statistical power of 80% a sample size of 182 patients is needed (given a two-sided alpha value of 0.05). The sample size calculation does not account for the stratified randomization [31]. To compensate for an expected loss of follow-up in approximately 5% of the patients, a total of 192 patients are included. Sample size calculation was performed using commercially available statistical software (Stata version 12, StataCorp, College Station, TX, USA) to estimate the sample size for a two-sample comparison of proportions.

### Randomization and blinding procedure

Patients are randomized using a randomization module in the eCRF. An external data manager is responsible for the eCRF, including the randomization module. A random allocation sequence is created using a standard computerized random-number generator. Patients are randomized in a permuted-block design employing different sized blocks. The randomization is stratified according to presence or absence of coronary artery disease (≥50% stenosis of one or more major epicardial arteries as revealed by a recent invasive coronary angiography or medical records documenting a previous myocardial infarction. Invasive coronary angiography is performed before CRT implantation if no recent examination (within two years) is available). The stratified randomization ensures a balance between treatment groups with 50% of patients with coronary artery disease in each group. Study subjects are allocated 1:1 to each intervention arm. Physicians responsible for enrollment of patients have no knowledge of block size or allocation sequence. Information on randomization is available in the implant window of the eCRF. Only the implanting physician has access to this window. When the patient is randomized to imaging guided LV lead placement the implant window contains uploaded images illustrating the LV region with the latest mechanical activation, distribution of scar tissue, and cardiac venous anatomy, respectively. When randomized to empiric LV lead placement the implant window will not show these images; thus, the implanting physician does not have access to information on LV mechanical activation pattern, distribution of scarred myocardium, and coronary venous anatomy prior to CRT implantation.

All baseline and follow-up evaluations, including clinical assessment, image acquisitions and analyses, are blinded with respect to randomization. Patients are blinded to the intervention arm. Randomization data will be available when all patients have completed follow-up.

### Statistical analysis

Demographic and baseline characteristics of both intervention groups are presented and compared clinically. At follow-up, a Pearson’s *χ*^2^ or Fisher’s exact test when appropriate is used to compare categorical variables. Normally distributed continuous variables are compared using Student’s *t*-test. Continuous variables not normally distributed are compared using a Wilcoxon’s rank-sum test. Forty patients will be randomly selected for assessing intra- and interobserver variability for the dyssynchrony measurements using linear regression and Bland-Altman analysis. In the same 40 patients, kappa statistics are computed to assess intra- and interobserver agreement of site with latest mechanical activation and final LV lead position. All analyses will be conducted according to the intention-to-treat principle. Multiple imputation will be applied in case of missing data [32]. A two-sided *P-*value of <0.05 is considered to be statistically significant. Statistical software (Stata version 12, StataCorp, College Station, TX, USA) is used for statistical analysis.

## Discussion

The introduction of CRT has improved the treatment of heart failure patients [1-4]. However, 30 to 50% of patients continue to remain non-responsive to this pacing strategy [1,2,4]. Optimal placement of the LV pacing lead has been shown to be a crucial determinant of CRT response [6-8]. The ImagingCRT study is a randomized trial designed to test the hypothesis that multimodality imaging guided LV lead positioning can increase the response rate to CRT as compared with standard LV lead placement.

Several retrospective echocardiographic studies using speckle tracking demonstrated greater reverse LV remodeling and superior long-term prognosis in patients with LV pacing concordant to the site of latest mechanical activation when compared to patients with discordance between LV lead position and the area with latest activation [7,10,26]. Accordingly, in this study, assessment of the latest mechanically activated LV segment is performed using speckle tracking 2D radial strain.

Recent studies used speckle tracking low-amplitude LV radial strain as a surrogate measure of myocardial scar to demonstrate a poor CRT response when the LV lead was placed in regions with low-amplitude radial strain [7,33]. A 2012 assessor-blinded, prospective randomized study found increased response rate to CRT using speckle tracking radial strain to assess the site of latest activation and as a measure of scar to guide LV lead placement as compared to standard therapy [16]. However, low-amplitude radial strain may also occur in non-ischemic heart failure patients with dilated LVs without myocardial scar [34]. Retrospective magnetic resonance imaging (MRI) and SPECT studies have demonstrated an unfavorable outcome after CRT when the LV lead is positioned in areas with transmural myocardial scar [6,9,12]. Myocardial scar quantification by SPECT is well validated in the literature and a practical approach for current clinical practice [14]. Consequently, SPECT imaging is performed for localization of myocardial scar in the current study. The LV 17-segment model is used for optimal agreement with echocardiography and cardiac CT [29].

Visualization of cardiac venous anatomy by cardiac CT is feasible and comparable to venography [15]. Cardiac CT can reveal the presence of specific CS tributaries and provide information on vessel course, side branches and diameters prior to CRT implantation. Thus, cardiac CT may further facilitate LV lead positioning to the optimal pacing site. Therefore, in this study, a cardiac CT is performed prior to CRT implantation.

Prior studies evaluating the effect of LV lead position on outcome after CRT have used post-implantation chest radiography, some combined with fluoroscopy, to determine final LV lead position [7,9,10,16,33]. However, chest radiography and fluoroscopy are poor predictors of LV lead position compared to cardiac CT [35,36]. Accordingly, we apply cardiac CT for assessment of LV lead position.

The criteria for response to CRT used in both prospective and retrospective studies investigating the effect of LV lead placement have been variable and often limited to changes in LV systolic dimension [7,10,16]. In contrast, we apply a robust composite endpoint combining subjective and objective derived measurements representing a global assessment of the patient [37].

### Limitations

We acknowledge the inherent limitations of the single-center study design. Nevertheless, this is a relatively large prospective, patient- and assessor-blinded, randomized study evaluating the effect of multimodality imaging guided LV lead placement on the response rate to CRT.

We do not merge the different imaging modalities. However, visualization of CS tributaries by cardiac CT can easily be related to the clockwise method for fluoroscopic CS visualization, which, again, may be correlated to the LV segmentation used for myocardial SPECT and echocardiography, respectively. Another limitation is the lower spatial resolution of scar quantification by SPECT as compared to MRI. However, a substantial minority of our patients is not eligible for MRI because of an indwelling device.

Because of the radiation exposure associated with, for example, cardiac CT and SPECT, there has been much scrutiny on the increasing use of diagnostic cardiac imaging [38]. Several approaches to minimize radiation dose are applied in this study, including the use of iterative reconstruction algorithms, application of prospectively triggered high pitch CT scan, individual settings of tube voltage and current, tube current modulation with narrow full current window, and the use of ^99m^Tc-agents in SPECT, respectively [39]. However, the introduction of cardiac CT and SPECT into the diagnostic algorithm prior to CRT will increase the cumulative radiation exposure of patients. In the age group investigated in this study, however, the stochastic risk of radiation-induced cancer may likely be outweighed by the potential benefit of multimodality imaging guided LV lead placement in CRT.

### Perspective

Despite tremendous advances in knowledge and experience with CRT, the proportion of patients considered clinical non-responders have remained stable since the introduction of CRT. The effect of imaging guided LV lead placement on CRT response rate, as tested in this study, can make an important contribution in the pursuit of increasing response rate to CRT.

### Trial status

The trial is ongoing. The first patient was randomized on 11 April 2011. As of August 2012, 88 subjects had been included. Enrollment completion is expected by September 2013.

## Abbreviations

2D: Two-dimensional; 3D: Three-dimensional; 6MWT: Six-minute-walk test; AV: Atrioventricular; CRT: Cardiac resynchronization therapy; CRT-D: CRT-implantable cardioverter defibrillator; CRT-P: CRT pacemaker; CS: Coronary sinus; CT: Computed tomography; eCRF: Web based case record form; EDV: End-diastolic volume; EF: Ejection fraction; ESV: End-systolic volume; LAO: Left anterior oblique; LBBB: Left bundle branch block; LV: Left ventricle/ventricular; MRI: Magnetic resonance imaging; NT-ProBNP: N-terminal pro b-type natriuretic peptide; NYHA: New York Heart Association; RAO: Right anterior oblique; RV: Right ventricle/ventricular; SPECT: Single-photon emission computed tomography; TDI: Tissue Doppler imaging; VV: Interventricular.

## Competing interests

The authors declare that they have no competing interests.

## Authors’ contributions

AS, MBK and JCN have contributed to conception and design of the study. All authors are contributing to acquisition of data and were involved in drafting and critical revision of the manuscript. All authors have read and approved the final version of the manuscript.
